# Cervical cancer screening program in Indonesia: is it time for HPV-DNA tests? Results of a qualitative study exploring the stakeholders’ perspectives

**DOI:** 10.1186/s12905-024-02946-y

**Published:** 2024-02-16

**Authors:** Fitriana Murriya Ekawati, Putri Listiani, Sri Idaiani, Jarir At Thobari, Firdaus Hafidz

**Affiliations:** 1 Department of Family and Community Medicine, Universitas Gadjah Mada, Yogyakarta, Indonesia; 2 Centre for Health Financing Policy and Health Insurance Management, Universitas Gadjah Mada, Yogyakarta, Indonesia; 3 Research Centre for Preclinical and Clinical Medicine, National Research and Innovation Agency Indonesia, Jakarta, Indonesia; 4 Indonesian Health Technology Assessment Committee, The Indonesian Ministry of Health, Jakarta, Indonesia; 5 Department of Pharmacology and Therapy, Universitas Gadjah Mada, Yogyakarta, Indonesia; 6 Department of Health Policy Management, Universitas Gadjah Mada, Yogyakarta, Indonesia

**Keywords:** Cervical cancer, Screening, HPV-DNA, Low-and-middle-income countries, Indonesia

## Abstract

**Objectives:**

Cervical cancer is a significant public health concern in Indonesia, and effective screening methods are necessary to improve the detection and reduce mortality. This study aimed to explore the perspectives of high-level stakeholders involved in cervical cancer screening in Indonesia and whether the use of human papillomavirus-deoxyribonucleic acid (HPV-DNA) methods for cervical cancer screening is acceptable in Indonesian settings.

**Methods:**

A qualitative research approach guided by realist evaluation was applied using focus group discussions (FGDs) between December 2021 and February 2022, conducted with stakeholders involved in cervical cancer screening in Indonesia. They were representatives of practitioners and policymakers involved in the screening, and were recruited through purposive recruitment. The data were analysed using inductive approach of thematic analysis.

**Results:**

The analysis of two FGDs with 29 participants identified four main themes: (i) Visual inspection with acetic acid (VIA) method as the most common modality used for cervical cancer screening, (ii) the applied screening programs in the community, (iii) the perceived challenges on the screening program, such as limitations of the National Health Insurance benefits package and a lack of regulations regarding screening procedures, and (iv) the possibilities of HPV-DNA testing as another modality for cervical cancer screening. Incorporating HPV-DNA testing also needs to ensure the capacity of the workers and the readiness of healthcare facilities.

**Conclusion:**

Although HPV-DNA testing is a promising modality, challenges on the cervical cancer screening in Indonesia remain on the coverage, accessibility of the tools in practice and the women’s awareness towards the screening. Ultimately, the findings of this study would help inform policies to improve cervical cancer screening programs in Indonesia.

**Supplementary Information:**

The online version contains supplementary material available at 10.1186/s12905-024-02946-y.

## Introduction

More than 90% of cervical cancer cases and deaths occur in low- and middle-income countries (LMICs), including Indonesia [1]. Unfortunately, many women diagnosed with cervical cancer in the setting have a more unfavourable prognosis due to detection at advanced stages and worsened with the limited management options at the healthcare providers [2, 3]. Although Indonesia has made some progress since the 2018 report on the Indonesian breast and cervical cancer implementation [2], there are still numerous reasons for concern; Indonesia is ranked number 2 among other Asian countries in terms of mortality and incidence of cervical cancer with age-standardised incidence (ASIR) 23,4 per 100.000 women, and age-standardised Mortality Rate (ASMR) 13,9 per 100.000 women. While the World Health Organization (WHO) suggested reducing cervical cancer ACIR to less than 4 per 100.000 women through vaccination, screening, precancer treatment [4], and encouraging women aged 35–45 to be screened; the coverage for cervical cancer screening in Indonesia was only 9,8% of the target population [2], far from the WHO recommendation of 70% screening coverage [4, 5]. Therefore, the screening is becoming more crucial to provide better care for women, onsidering cervical cancer may take years to develop, which thus detection at early stages will result to better prognosis [6].

Meanwhile, despite of their importance, the modalities used in the cervical cancer screening in Indonesia are limited. The usual modalities for cervical cancer screening are visual inspection using acetic acid (VIA), Papanicolaou Smear (PAP Smear), colposcopy, and recently introduced Human Papilloma Virus (HPV)-DNA tests; with the most widely used test in Indonesia is VIA, with a target population of those women aged 30–50 years old, and funded with the government public health scheme [7–10]. The uptake of the screening was also challenged by many factors attributed to the procedures, such as the providers’ limited capability, limited access to the program and the women’s hesitance regarding the procedures [11, 12].

Meanwhile, literature suggests that HPV DNA tests have the potential to be the most effective screening tool for cervical cancer, surpassing the accuracy of Pap Smears for detecting cervical intraepithelial neoplasia grade 2 (CIN2+) [13–15]. A high-risk non-genotyping HPV DNA test demonstrated a sensitivity of 81.5% and specificity of 91.6% [13, 14], while Pap Smear sensitivity and specificity were reported as 58.4% and 87.7%, respectively [15, 16]. In terms of detecting CIN 3+, HPV DNA with high-risk non-genotyping has 85,3% sensitivity and 91% specificity, but Pap Smear test has 62.7% of sensitivity and 87.4% of specificity [13, 14]. The WHO then also provides policy recommendations on the use of the HPV DNA test as a choice for primary screening for cervical cancer [5]. However, in Indonesia, current national health insurance does not cover for HPV-DNA examination. It only covers VIA and Pap smear cervical cancer screening inside the primary health care centre buildings with referrals for further treatment if necessary [17].

Until now, studies about cervical cancer screening policies using HPV-DNA in the Indonesian context are also limited. Even though various strategies have been implemented as above [2] and with the addition of the Minister of Health regulations number No. 29 of 2017 [18] and JKN regulations No. 2 of 2019 [19], the endorsement of the use of HPV DNA is lacking. To respond to this situation, there is a need to understand the high-level stakeholders’ perspectives regarding the implementation of cervical cancer screening with various modalities in Indonesia and whether it is appropriate to take the HPV-DNA tests as another alternative to the cervical cancer screening. Our study was part of a more extensive study aiming to determine the possibility of adopting HPV-DNA tests as one of the screening modalities for cervical cancer screening in Indonesia from the Indonesian Ministry of Health 2021. This qualitative exploration then aimed to explore the supply-side stakeholders’ perspectives on cervical cancer screening in Indonesia and whether HPV-DNA tests are acceptable to be adopted as national cancer screening modalities.

## Materials and methods

### Design and theoretical framework

This qualitative study adopted the realist evaluation approach as the guiding theoretical framework. The approach provides guidance for evaluating health care intervention, particularly to explore how an intervention has been performed and what conditions affecting the implementation process in practice [20, 21]. In the context of this research, the realist evaluation helped the authors to provide a description and evaluation of the current situation of cervical cancer screening in Indonesia, and the potential adoption of HPV-DNA as a modality for mass cervical cancer screening, considering factors supporting and challenging its implementation.

In line with the selected theoretical framework, our study applied qualitative study using focus group discussions (FGDs) as the data collection method. This method enables a flexible yet comprehensive discussion on the topic and a variety of answers gained from the participants. The method also allows participants to interact dynamically, and stimulate more ideas, perspectives and discussions about the topic [22, 23].

### Participants and recruitment

Our study participants were high-level key stakeholders involved in cervical cancer screening in Indonesia. They were representatives of the Indonesian Ministry of Health officers, representatives of local health officers at both provincial and district levels, representatives of national insurance body officers (BPJS-K: *Badan Penyelenggara Jaminan Sosial-Kesehatan*), the representatives of professional organisations, such as medical doctors, midwives and obstetricians, and those obstetrician consultants. They were identified from the authors’ professional networks collaborated with one of the directorates in the Indonesian Ministry of Health, and were recruited through an invitation to participate in the study from November to December 2021. The prospective participants were sent information and invitation to participate in the study, plain language statements, and consent forms, and were informed that their participation was voluntary. The recruitment process was conducted from the mailing lists of professional networks from the Indonesian Ministry of Health departments related to this study, such as the public health division, health service division, local health officers networks, and networks for professional organisations. Those who agreed to participate were invited to attend the FGDs. All of the participants had provided written consent to participate in this study.

### Data collection

Two authors, academic researchers and researchers from the Ministry of Health conducted the data collection. Participants who agreed to participate were grouped based on their background and professional occupation. The first group consisted of clinicians and professional organisations, such as the representatives of obstetrician and midwifery organisations, while the other group consisted of policymakers, such as representatives of the Ministry of Health, the insurance office and local health officers. The data collection process was conducted online using Zoom teleconference [24] from December 2021 to February 2022 and being recorded. The online FGDs were selected to anticipate COVID-19 restrictions in Indonesia while this study was conducted.

During FGDs, the participants were asked using guiding questions related to cervical cancer screening implementation in Indonesia, including (i) their views and experiences for cervical cancer screening modality, (ii) facilitators and (iii) challenges of current screening program, and (iv) the potentials and challenges of using HPV-DNA as a modality for cervical cancer screening in Indonesia. These guiding questions were previously tested and received feedback during initial FGDs with a mock group of participants.

### Data analysis

The data obtained from the FGDs were analysed thematically using the following steps:

(i) The FGDs recordings were transcribed, and (ii) all of the transcripts were then uploaded into NVIVO software version 12 [25], and were read repeatedly to achieve data familiarisation. (iv) The data were then coded for any notable yet significant quotes and grouped into themes and overarching themes [26]. All authors validated the coding process, and the results were discussed in a series of meetings until a consensus on the data analysis was reached. To ensure the credibility of the study, this manuscript follows the guidance of reporting a qualitative study using Standards for Reporting Qualitative Research (SRQR) [27].

### Trustworthiness of the study

To ensure the trustworthiness of the study, we applied strategies as follows. Researchers in the study are academic physicians, health economists and researchers from the Ministry of Health of Indonesia with extensive experience involved in disease screenings, including cervical cancer. The guiding questions for the FGDs were previously tested between the researchers and a mock group of participants. The study design was also previously consulted to several research consultants provided by the Ministry of Health, who were obstetricians and academic researchers with extensive experience of cervical cancer screening, research design and qualitative methods. Questions performed for the interviews and considerations of the expected answers were also consulted with these consultants during the mock activities and the data analysis. Several meetings between the researchers were also conducted to ensure similar interpretations of the participants’ quotes. The presentation of the themes during the analysis was also equipped with supporting quotes from the participants [28, 29].

## Results

We conducted two focus group discussions (FGDs) with key representatives of Indonesian cervical cancer screening policy stakeholders. The first FGD had 13, while the second had 16 participants, with discussions lasting from one to two hours. The stakeholder participants represented national-level organisations such as the Indonesian Ministry of Health’s Directorate of Prevention and Control of Non-Communicable Diseases, the Center for Health Financing Policy and Decentralization, and the national health insurance (BPJS-K). They also included representatives from professional organisations such as oncology gynecologists, midwives, and physicians working in various screening centres. The first FGD had ten female and three male participants from professional organisations, while the second FGD had 11 female and five male participants who were policymakers. Most participants were medical doctors (*n* = 15), while others were public health practitioners, health economists, and midwives (Table 1).

**Table 1:** Summary of the participants’ characteristic

Characteristics of participants	Number (n)
**Sex**	
Male	8
Female	21
**Institution** (participants can tick more than one options)	
BPJS Kesehatan	2
District Health Office	3
Ministry of Health	16
Profession organisation	8
**Educational Background** (participants can tick more than one options)	
Health Economist	6
Medical doctor	16
Midwife	1
Public Health	6
**Age**	
30–39	12
40–49	12
50–59	4
60–70	1
**Working Experiences**	
> 15 years	7
11–15 years	12
5–10 years	10
**City of Residence**	
DI Yogyakarta	1
DKI Jakarta	20
East Java	1
West Java	7

The study participants primarily originated from Jakarta, West Java, Yogyakarta, and East Java provinces, including representatives from the Ministry of Health in Jakarta. These provinces were represented by clinicians or gynaecologist oncology from the tertiary hospital and District Health Office. Whilst the central government participants were from the Ministry of Health of Republic Indonesia, professional organisation, and the national insurance office (BPJS-K). All of the participants were actively involved in the screening and the clinical and public health aspects of cervical cancer screening, including their familiarity with cervical cancer screening using HPV-DNA tests in their regions.

Our analysis elicited four themes related to the stakeholders’ perspectives on cervical cancer screening in Indonesia as follows:

### Current screening modalities used in practice 

Almost all participants in the two FGDs perceived that cervical cancer screening modality that was widely used in Indonesia was the VIA method. This method was mentioned as affordable, fast and widely available in almost all health services, including all health centres in rural and remote areas in Indonesia. The use of VIA has also been carried out extensively and collaboratively between midwives, doctors and obstetricians across Indonesian provinces. Moreover, VIA examinations in some areas has been double-crosschecked and and validated using remote consultation whenever requested by the first examiner, so that the examination results could have higher sensitivity and specificity. This remote-validation mechanism is called TeleDoVia.

“*The cervical cancer screening in Indonesia mostly is conducted using VIA methods, but the VIA examinations is also equipped with TeleDoVia, which is an VIA examination that is validated by remote consultation with an obstetrician, here, the sensitivity results obtained are higher than the casual VIA*” (FGD 1, Participant 1).

The second most widely used cervical cancer screening modality for cervical cancer screening in Indonesia is PAP Smear. This method is carried out collaboratively between clinicians by taking samples that are usually conducted at primary care level, with smears being analysed by pathologists in hospitals or private laboratories.

“*In our opinion, VIA is still the first choice for screening of cervical cancer (in Indonesia). Even though for us in Jakarta, the health facilities are quite complete and laboratories are also available, we also have to consider those in rural areas, those who are far from health facilities or those who are far from a PA (pathologist) expert, where if they do Pap Smear, the specimen must be sent to the district hospital and it takes time. However, VIA is an examination that can be done extensively, and the results can be obtained directly. Therefore, in terms of costs, it is very cost-effective because it is cheap and the tools are simple, (Examinations in) Jakarta also already use disposable speculums, but maybe other areas are not yet (using) disposable tools*.” (FGD 2, Participant 1).

### Applied screening programs

As cervical cancer screening in Indonesia is part of public health programs, many mass-screening activities have also been widely held in the community i.e., the VIA screening program. Many participants mentioned and agreed that cervical cancer screening is a priority procedure for women of reproductive age with a history of sexual activities. The VIA screening target in Indonesia is also high and is always promoted by many stakeholders at the national and regional levels as an effort to prevent cervical cancer.

“*Then, regarding how to promote the uptake, if we look at the principle, not a single woman should miss it as it is a basic service (for screening), meaning everyone must receive it. Our target is also very high, up to 100% of the women. Unfortunately, we limit (the screening) to women aged 30–50 and who already have a history of sexual intercourse. That is indeed the limitation in the program, included in the screening for breast and cervical cancer*” (FGD 2, Participant 2).

“*In 2017–2018, we carried out a very ambitious VIA screening target across Indonesia, our participants reached 93,000 women in a one-day event, and we had 2,500 women who had lesions and were then referred to the nearest primary care service. We also achieved an international record then*” (FGD 1, Participant 2).

Moreover, many health workers, especially midwives and doctors, have been trained to perform VIA by the Indonesian obstetricians and midwifery association. The training also includes further innovations to support the program, such as the involvement of cadres/community health workers (CHW) and VIA competition to expand the coverage of cervical cancer screening:

*“There have been many trainings conducted from the obstetrician associations to support the VIA screening” (FGD 1, Participant 1).*

*“We also involve the cadres and meetings for women. We also held an annual VIA innovation competition. We bring innovation from cadres to educate the women as our target community, especially those in childbearing age, to participate in the (screening) VIA examination” (FGD 2, Participant 4).*

### Perceived challenges of cervical cancer screening in Indonesia

Despite the extensive targets and supporting factors above, there are several challenges to cervical cancer screening in practice, including the effect of the COVID-19 pandemic, limited national insurance reimbursements and restricted government regulation. The first challenge mentioned by some participants was the temporary effect of COVID-19 pandemic; that the restrictions resulted in many women being prevented from coming to health facilities for non-essential services, and cancelled mass screening events that were previously available in the community clinics.

*“A few years ago, in 2017 or 2018, our achievements were quite high because there were regulations took place to support the program, but during this pandemic, we experienced a declining participation because (of the restriction) not everyone is willing to come to the health facility as they are afraid of catching the COVID, and the restriction itself prevent us to hold mass-screening activities in an event " (FGD 2, Participant 1).*

The second challenge is the national insurance claims and reimbursement, which also limit the scope of cervical cancer screening. This limitation is primarily for VIA financing, which cannot be covered by the national insurance in Indonesia (BPJS-K) as it is covered through different funding scheme from the Ministry i.e. the public health or non communicable disease funding. If the screening is carried out outside the health facility, it can not be covered by the insurance as it is prone to potential double claims with the government funding. However, if the examination is carried out inside a health facility, the insurance could reimburse the performed examination, but is limited to only a few tests per year. On the other hand, even though it is less popular among women, the insurance can claim Pap Smear because the procedure is usually carried out inside a health facility. While HPV-DNA is not yet covered by JKN at the moment, and is only available in private laboratories/practice as it is not yet considered as routine examination for mass-screening program in the community.

*" Puskesmas (community health centers) can claim for those with Pap smears if the examination is conducted inside the health facility buildings, but those for VIA cannot be claimed (to BPJS-K) because it is usually conducted in the community (outside the health facilities)” (FGD 2, Participant 6).*

*“There is a regulation from BPJS-K that the VIA test can be claimed if it is done at the health facility. When the screening is conducted outside the health facility building, the Puskesmas building, or in the middle of community event, it can not be covered by the insurance but by the public health programs from the health office.” (FGD 2, Participant 1).*

In addition to the above claims and pandemic barriers, the third challenge is the government regulations on cervical cancer screening, which does not provide sufficient guidance regarding the implementation of screening in the field. There is also no detailed guidance regarding medical procedures that can be performed, whether using VIA, or Pap Smear or HPV-DNA; and many health offices in the regions are developing different policies for the screening program as part of the non-communicable disease screening scheme to allow more mass-screening programs, here using VIA methods as the most affordable methods.

*“The medical service standard does not explicitly mention cervical cancer screening, in Jakarta, we include it (the program) in the non-communicable disease risk factor screening, but for age range is for women 30–50 years old, with a little note those are only for married women or those already had sexual intercourse and using VIA tests” (FGD 2, Participant 1).*

Meanwhile, the fourth challenge, there is also still a need to train health workers and strategies for improving community awareness of cervical cancer screening, particularly training for the newly graduated general practitioners (GP) as they might only have a relatively short training at the university. While often, women also feel reluctant to the screening procedure, i.e. pelvic examinations, afraid of the examination results, then feel shy and conclude that there is no need to take part in a VIA or PAP Smear examination. Several FGD participants then proposed alternatives to increase patient participation in this examination, including strengthening cross-sectoral collaboration and accommodating this examination at the same visit when the patient is seeking treatment, for example, during family planning services.

*“Talking with the association of medical education institutions, GPs are legitimate to do the screening, that it is true that they have the authority and ability to perform the procedure, but in reality, there was only a little opportunity for them to learn the procedure during their training at the university, and there were only a few cases (screening) they met, so any training is always needed to remind them (the GPs) about VIA and Pap Smear procedures” (FGD 2, Participant 6).*

*“We once conducted an evaluation with women (for the low screening uptake), and one of the reasons was their fear with the procedures and results, and feel shy when they were going to have a VIA because not everyone felt comfortable with the procedures, also they were afraid and anxious with results” (FGD 2, Participant 5)*.

“*Everyone has to participate in the cancer screening; most of the ‘troops’ are midwives, but midwives alone are not enough. We need more collaborations with other ministries such as the Ministry of Women’s Empowerment, the Ministry of Home Affairs, and others (FGD 1, Participant 3 and 1).*

### The possibilities of HPV-DNA as a modality for mass screening in Indonesia 

Participants in the study also discussed the possibility and potential of using the HPV-DNA test as a primary cervical cancer screening modality in Indonesia. Participants acknowledged that the HPV-DNA test had a higher sensitivity and specificity, and its potential as a self-administered test. One of the participants also proposed that combining HPV-DNA with VIA or Pap Smear has a high sensitivity level of up to more than 90%. The HPV-DNA test is also considered to have a competitive price due to its higher sensitivity level.

“*Indeed, most of them even say 70%, some say above 90% of cervical cancer is caused by HPV infection so that if using HPV DNA, we can detect HPV infection earlier as the cause of cervical cancer. So, of course, this is an excellent examination method to see the possibility of cervical cancer in the future” FGD 2, Participant 7).*

*“We now try to propose for HPV-DNA test costs to 178 thousand rupiahs per test, although it is still more expensive than the Pap Smear, which is only 125 thousand rupiah per test (FGD 1, Participant 1).*

*“HPV-DNA can be used to increase coverage, self-sampling can be promoted, but more enforcements are still needed to increase the low uptake cervical cancer screening” (FGD 1, Participant 4).*

Meanwhile, participants also noted that implementing the HPV-DNA examination would have several crucial challenges. Lack of awareness about the test is a significant barrier, as many clinicians in primary healthcare facilities are unfamiliar with it. Additionally, the availability of HPV DNA testing is lacking across Indonesia, highlighting the need for more supply-side efforts.

“*Then, for HPV-DNA itself, we have never been exposed to it even though I know we have had research from a university if I am not mistaken, but until now, it has never been administered or carried out for follow-up in primary care level.” FGD 2, Participant 5).*

*“Implementation means how many health facilities throughout Indonesia have it, and how many are capable of providing and performing HPV-DNA, which might be a consideration for us, we can not adopt it to become a screening modality when we know the fact that there are still many areas that have not been able to provide HPV-DNA tests (due to availability)” ( FGD 2, Participant 6).*

Returning to the framework we used to guide the study using the realist evaluation approach [20, 21], the participants’ quotes were then incorporated to provide the explanation of the cervical cancer screening program and the possibilities of the uptake of HPV-DNA as another screening modality in Indonesia (Table 2).

**Table 2:** Summary of the realist evaluation domains: context, mechanism and outcome for the cervical cancer screening in Indonesia

Context	Mechanism	Outcome
a. Current screening modalities, including VIA and Pap smear, are cost-effective and widely available. b. Applied screening programs are part of public health efforts, targeting sexually active women in reproductive ages. c. Perceived challenges in implementing cervical cancer screening include the COVID-19 pandemic, limited national insurance reimbursements, and government regulations. d. Uneven distribution and lack of awareness of the HPV-DNA test nationwide.	a. Stakeholders acknowledge the potential benefits of the HPV-DNA test, including higher sensitivity, specificity, and self-sampling capabilities. b. Training and capacity building for healthcare professionals, especially general practitioners, in cervical cancer screening, including HPV-DNA tests. c. Cross-sectoral collaboration and integrating cervical cancer screening into other healthcare visits (e.g., during contraceptive visits) to improve participation and coverage.	a. Mass screening improved cervical cancer screening coverage and detection rates, potentially reducing the burden of cervical cancer in Indonesia. b. Increased awareness and utilisation of HPV-DNA tests as a screening modality, leading to more accurate and screening of cervical cancer cases. c. Implementation of HPV-DNA testing as a standard screening modality, contingent upon overcoming barriers such as lack of awareness, uneven distribution, and government regulations.

## Discussion

This research explored the way and progress of cervical cancer screening, including the possibilities of HPV-DNA to be included as a screening modality for cervical cancer in Indonesia. Our results reported views and perspectives from high-level key stakeholders related to the screening program, that the VIA method is still the most accessible screening procedure implemented widely and complemented with many initiatives to support the program. However, the cervical screening itself faced challenges during the COVID-19 pandemic and patients were still reluctant to take up the program due to their hesitance in the procedures. While on the other side, even though HPV-DNA is promising to be taken as another mass screening modality, challenges are still in its nationwide availability, affordability, and supplies in practice.

Our findings support previous research of the most accessible and convenient screening method using VIA in Indonesia [7]. Despite this convenient access, the cervical screening coverage in Indonesia remains limited, particularly among women with lower education and income levels due to imited support from spouses, limited knowledge and skills of healthcare providers [30, 31]. The availability of screening tests also varies across different regions of Indonesia, with greater access to mass screening in urban areas such as in the provinces in Java and Bali, while provinces such as Papua, West Papua and East Nusa Tenggara have further significant limitations regarding screening tests availability [2]. Additionally, the COVID-19 pandemic has posed another challenge in practice due to restrictions on mass-gathering and clinic attendance [32, 33].

Using the realist evaluation framework [20, 21], this study also highlights on the process and evaluation of cervical cancer screening in Indonesia. VIA examination is the most widely used modality for cervical cancer mass screening, which is usually conducted as part of public health programs in primary care, targeting sexually active women in their reproductive ages. While other tests, such as Pap Smear and HPV-DNA, are considered to have higher sensitivity, specificity and the HPV DNA opportunity for self-sampling capabilities, their wide availability across Indonesia is still limited. Nonetheless, the significant challenge of the screening program in Indonesia are not only the modalities,but also the screening coverage itself. Therefore, the attention of the public health intervention should primarily aim to reach the extensive screening using any available modalities. The mass-screening program then needs to always be promoted, as well to promote their benefits to prevent severe morbidities and mortalities related to cervical cancer [6, 30, 34].

Our study has some practical and policy implications. First, our findings suggest more efforts to correspond to the limited access and coverage of cervical cancer screening that has been paused by the COVID-19 pandemic, including supporting policies, financing of the program by the national insurance and the importance of promoting the screening to women. Healthcare providers must also be better equipped with appropriate skill training to perform and educate their patients effectively. Secondly, policymakers need to consider the disparities in access to screening across different regions in Indonesia, with particular attention to improving access and participation in under-resourced areas. Finally, given the potential of HPV-DNA tests to improve sensitivity, specificity and the possibility of self-administered modality in cervical cancer screening, further research is needed to promote the availability and affordability of the test, as well as the users’ views of the possibility of HPV-DNA self-administered tests. By addressing these issues, policymakers and healthcare providers are expected to improve screening coverage and ultimately prevent cervical cancer in Indonesia.

Our research is not without any limitation. Firstly, the study was conducted during the peak of the COVID-19 pandemic in Indonesia in late 2021–2022, and the focus groups were conducted online, which might have limited the dynamic discussion among the study participants. Secondly, the sample size was limited, even though we have engaged representatives from the government and various prominent professional associations involved in cervical cancer screening in Indonesia, such as the Ministry of Health, local health offices, general practitioners and midwifery associations. Thirdly, most of our stakeholder participants were based in Java Island, and we have not yet covered the views of women, who might have different views, experiences, and expectations toward cervical cancer screening. Therefore, careful consideration is needed to generalise our findings in Indonesia.

Our study also implies further research and policies to strengthen and endorse the implementation of cervical cancer screening in Indonesia, due to that cervical cancer is still one of the most frequent cancers for women in Indonesia, and the patients are usually detected in late stages [3]. Further research is needed to explore possibilities for the extensive availability of all screening modalities, advocating for a better financing system for cervical screening, including a lower price and the potential of co-testing HPV-DNA with VIA or Pap Smear, to significantly increase the screening coverage. Even though the availability of HPV-DNA is still limited in private sectors; with more supplies and endorsement, the test can potentially be considered for mass screening due to its benefits of self-collection process. However, this process may also need more discussion on financing this program of this program due to the self-administered test being conducted outside the facility. As well there is a need to conduct a study on the appropriateness of primary HPV testing with or without combining it with cytology or VIA. Also to explore the views and expectations from women as the ultimate users of the screening program. These strategies are expected to enable women to have a more effective and higher sensitivity procedures for cervical cancer screening in Indonesia.

## Conclusion

Cervical cancer screening in Indonesia is a priority program for Indonesian sexually active women, with VIA as the most used method for the program. However, challenges of the screening in practice remain on the access, supporting policy, lack of awareness of the women, and restrictions due to the COVID-19 pandemic. While HPV-DNA testing is a promising procedure with a higher level of sensitivity, its uptake as a mass screening procedure in Indonesia is again, challenged by its limited availability and affordability in practice. Further research is needed to strengthen the policies, and advocate for the supply and affordability of HPV-DNA tests as another potential modality for cervical cancer screening in Indonesia.

### Electronic supplementary material

Below is the link to the electronic supplementary material.

Supplementary Material 1

## Data Availability

Data generated from this study are not publicly available due to maintain the participants? confidential identity and responses, however, are available from corresponding author (FE) on reasonable request.

## References

[1] Sung H, Ferlay J, Siegel RL, Laversanne M, Soerjomataram I, Jemal A (2021). Global Cancer statistics 2020: GLOBOCAN estimates of incidence and Mortality Worldwide for 36 cancers in 185 countries. CA Cancer J Clin.

[2] Wahidin M, Febrianti R, Susanty F, Hasanah SR (2022). Twelve years implementation of cervical and breast Cancer screening program in Indonesia. Asian Pac J Cancer Prevention: APJCP.

[3] Domingo EJ, Noviani R, Noor MRM, Ngelangel CA, Limpaphayom KK, Van Thuan T (2008). Epidemiology and prevention of cervical cancer in Indonesia, Malaysia, the Philippines, Thailand and Vietnam. Vaccine.

[4] Arbyn M, Weiderpass E, Bruni L, de Sanjosé S, Saraiya M, Ferlay J (2020). Estimates of incidence and mortality of cervical cancer in 2018: a worldwide analysis. Lancet Glob Health.

[5] Organization WH. WHO guideline for screening and treatment of cervical pre-cancer lesions for cervical cancer prevention. World Health Organization; 2021. 34314129

[6] Spagnoletti BRM, Atikasari H, Bennett LR, Putri HM, Rachellina M, Ramania A (2020). Hitting the pause button: the impact of COVID-19 on cervical cancer prevention, screening and treatment access in Indonesia. Asian Pac J Cancer Care.

[7] Utami TW, Nuranna L, Mahathir M, Peters AA, Fleuren GJ, Osse M et al. Visual inspection of Acetic Acid (VIA) as a Promising Standard for Cervical Cancer Screening. Indonesian J Obstet Gynecol. 2014:216–9.

[8] Vet J, Kooijman J, Henderson F, Aziz F, Purwoto G, Susanto H (2012). Single-visit approach of cervical cancer screening: see and treat in Indonesia. Br J Cancer.

[9] Nuranna L, Purwoto G, Hadisty A. EP372 DoVIA and TeleDoVIA as a documentation and consultation media in VIA methods of cervical cancer screening in Indonesia. BMJ Specialist Journals; 2019.

[10] Indarti J, Pratama YS. The accuration of liquid based cytology and HPV DNA test combination as precervical cancer lesion screening. Indonesian J Obstet Gynecol. 2017:241–5.

[11] Robbers GML, Bennett LR, Spagnoletti BRM, Wilopo SA (2021). Facilitators and barriers for the delivery and uptake of cervical cancer screening in Indonesia: a scoping review. Glob Health Action.

[12] Kementerian Kesehatan RI (2020). Profil Kesehatan Indonesia Tahun 2019.

[13] Ogilvie GS, Krajden M, van Niekerk DJ, Martin RE, Ehlen TG, Ceballos K (2012). Primary cervical cancer screening with HPV testing compared with liquid-based cytology: results of round 1 of a randomised controlled trial -- the HPV FOCAL study. Br J Cancer.

[14] Schiffman M, Herrero R, Hildesheim A, Sherman ME, Bratti M, Wacholder S (2000). HPV DNA testing in cervical cancer screening: results from women in a high-risk province of Costa Rica. JAMA.

[15] Koliopoulos G, Nyaga VN, Santesso N, Bryant A, Martin-Hirsch PP, Mustafa RA et al. Cytology versus HPV testing for cervical cancer screening in the general population. Cochrane Database Syst Reviews. 2017(8). 10.1002/14651858.CD008587.pub2 PMC6483676 28796882

[16] Jeronimo J, Bansil P, Lim J, Peck R. A multicountry evaluation of careHPV Testing, VisualInspection with Acetic Acid, and Papanicolaou Testing forthe detection of cervical cancerand the START-UP. Study Group International Journal of Gynecological Cancer; 2014. 10.1097/IGC.0000000000000084 PMC4047307 24557438

[17] Kesehatan B, Peraturan BPJS. Kesehatan Nomor 2 Tahun 2019 Tentang Pelaksanaan Skrining Riwayat Kesehatan dan Pelayanan Penapisan atau Skrininf Kesehatan Tertentu serta Peningkatan Kesehatan bagi Peserta Penderita Penyakit Kronis Dalam Program Jaminan Kesehatan Jakarta. 2019 [Available from: https://www.bpjs-kesehatan.go.id/bpjs/arsip/detail/1357.

[18] Indonesia KKR. Perubahan Atas Peraturan Menteri Kesehatan Nomor 34 Tahun 2015 Tentang Penanggulangan Kanker Payudara Dan Kanker Leher Rahim. 2017.

[19] Kesehatan B, JKN regulations No. 2 of 2019: Peraturan Badan Penyelenggara Jaminan Sosial Kesehatan Nomor 2 Tahun 2019 Tentang Pelaksanaan Skrining Riwayat Kesehatan dan Pelayanan Penapisan Atau Skrining Kesehatan Tertentu Serta Peningkatan Kesehatan Bagi Peserta Penderita Penyakit Kronis dalam Program Jaminan Kesehatan 2019 [Available from: https://peraturan.bpk.go.id/Home/Details/112083/permenkes-no-29-tahun-2017).

[20] Pawson R. Realist Evaluation. Adapted from Pawson R & Tilley N. Realist evaluation London SAGE. Available online at British Cabinet Office: London; 1997.

[21] Pawson R, Tilley N. Realist evaluation. 2004. Google Scholar. 2015:36.

[22] Ekawati FM, Emilia O, Gunn J, Licqurish S, Lau P (2020). The elephant in the room: an exploratory study of hypertensive disorders of pregnancy (HDP) management in Indonesian primary care settings. BMC Fam Pract.

[23] Ekawati FM, Emilia O, Gunn J, Licqurish S, Lau P (2021). Challenging the status quo: results of an acceptability and feasibility study of hypertensive disorders of pregnancy (HDP) management pathways in Indonesian primary care. BMC Pregnancy Childbirth.

[24] Inc ZVC. Zoom [Available from: https://zoom.us/team.

[25] International Q. Nvivo (Software) version 12.2.0 (3262). 2019.

[26] Patton MQ. Qualitative research & evaluation methods / Michael Quinn Patton: Thousand Oaks, Calif.: Sage Publications, c2002. 3rd ed.; 2002.

[27] O’Brien BC, Harris IB, Beckman TJ, Reed DA, Cook DA (2014). Standards for reporting qualitative research: a synthesis of recommendations. Acad Medicine: J Association Am Med Colleges.

[28] Gunawan J (2015). Ensuring trustworthiness in qualitative research. Belitung Nurs J.

[29] Ekawati FM, Claramita M (2021). Indonesian General practitioners’ experience of practicing in primary care under the implementation of Universal Health Coverage Scheme (JKN). J Prim Care Community Health.

[30] Setiawan D, Miranti I, Partiwi TD, Puspitasari DA, Ramadhan FN (2022). The willingness for cervical cancer screening among sexually active women in Indonesia: lesson learned from two districts. Int J Gynecol Obstet.

[31] Winarto H, Dorothea M, Winarno AS, Ibrahim NAA, Putri YM, Purbadi S (2022). Knowledge, attitude, and practice on cervical Cancer and HPV vaccination among medical students in Jakarta, Indonesia: a cross-sectional study. Open Access Macedonian J Med Sci.

[32] Winata IGS, Juniarti SE. Cervical Cancer Screening in COVID-19 Pandemic Era. 2020.

[33] Spagnoletti BRM, Bennett LR, Wahdi AE, Wilopo SA, Keenan CA (2019). A qualitative study of parental knowledge and perceptions of human papillomavirus and cervical cancer prevention in rural central Java, Indonesia: understanding community readiness for prevention interventions. Asian Pac J cancer Prevention: APJCP.

[34] Robbers GML, Bennett LR, Spagnoletti BRM, Wilopo SA (2021). Facilitators and barriers for the delivery and uptake of cervical cancer screening in Indonesia: a scoping review. Global Health Action.

